# Novel mechanisms of Collagenase Santyl Ointment (CSO) in wound macrophage polarization and resolution of wound inflammation

**DOI:** 10.1038/s41598-018-19879-w

**Published:** 2018-01-26

**Authors:** Amitava Das, Soma Datta, Eric Roche, Scott Chaffee, Elizabeth Jose, Lei Shi, Komel Grover, Savita Khanna, Chandan K. Sen, Sashwati Roy

**Affiliations:** 1 Department of Surgery, Center for Regenerative Medicine and Cell Based Therapies and Comprehensive Wound Center, The Ohio State University Wexner Medical Center, Columbus, OH USA; 2 Research & Development, Smith & Nephew, Inc., Fort Worth, Texas USA

## Abstract

Collagenases are useful in enzymatic wound debridement. Clostridial collagenase, marketed as Collagenase Santyl Ointment (CSO), is FDA approved for such use. Building on the scientific premise that collagenases as well as collagen degradation products may regulate immune cell function, we sought to investigate the potential role of CSO in wound inflammation. We tested the hypothesis that in addition to enacting debridement, CSO contributes to the resolution of persistent wound inflammation. Wound macrophages were isolated from PVA sponges loaded with CSO or petrolatum and implanted in mice. Significant increase in pro-reparative and decrease in pro-inflammatory polarization was noted in macrophages of acute as well as diabetic wounds. Wound macrophages from CSO-treated group displayed increased production of anti-inflammatory cytokines IL-10 and TGF-β, and decreased levels of pro-inflammatory cytokines TNF-α and IL-1β. The active ingredient of CSO, CS-API, induced the expression of mϕ^heal^ /M(IL-4) polarization markers *ex vivo*. CS-API treatment attenuated transactivation of NF-κB and significantly induced STAT6 phosphorylation. A significant role of a novel PGE2-EP4 pathway in CS-API induced STAT6 activation and the mϕ^heal^ /M(IL-4) polarization was identified. Taken together, findings of this work reposition CSO as a potential agent that may be effective in resolving wound inflammation, including diabetic wounds.

## Introduction

Chronic wounds are a major socioeconomic threat costing billions of dollars annually to the US healthcare system^1^. A critical aspect of chronic wound management is maintaining a clean and well-vascularized wound bed that can then successfully progress through the stages of wound healing^2^. Debridement of devitalized tissue from the wound bed is an essential component of wound bed preparation. Collagenase Santyl Ointment (CSO) is an FDA-approved clostridial collagenase based prescription ointment for effective enzymatic debridement of chronic wounds and burns^3^. CSO has been shown to be an effective adjunct to wound therapy^4,5^. While controlled expression of metalloproteinases, *e.g*. collagenases, is required for wound healing, persistent hyperactivity of endogenous metalloproteinases may be detrimental for healing^6,7^. Interestingly, CSO may have roles at the wound site that are beyond its role in debridement^8^. However, whether CSO activity at the wound site may modify the function of wound resident and immune cells remains unknown.

Unresolved persistent inflammation is a hallmark of chronic wounds^9,10^. Macrophages (mϕ) are one of the central players orchestrating the inflammatory response at the wound site^11,12^. Selective depletion of macrophages in the initial inflammatory phase of wound healing compromised angiogenesis and epithelialization^13,14^. Depletion of macrophages in the remodeling phase resulted in severe hemorrhage in the wound tissue^13^ pointing towards a key role of these cells in tissue repair. The removal of dead and dying cellular debris by mϕ from wounds is required for resolution of inflammation^15^. Mϕ show diversity and plasticity in structure and function^16^. The ‘classically activated’ mϕ are pro-inflammatory in nature and play a critical role in host defense against infection. The ‘alternatively activated’ mϕ are associated with tissue repair/remodeling and resolution of inflammation^16,17^. Recent studies indicate that these two phenotypes represent the two extremes of the full spectrum of mϕ functions/phenotypes^18^. Given the current ambiguity in macrophage nomenclature specifically for tissue macrophages^18,19^, and proposed misfit of wound macrophage (wmϕ) with the M1/M2 nomenclature^16,20,21^, for this work we classify *in vivo* wmϕ based on the pro-inflammatory (mϕ^inf^) or pro-resolution/healing (mϕ^heal^) polarization states. The *in vitro* polarized M1/M2 have been referred to as M(LPS + IFNγ)/M(IL-4) as per revised guidelines^18^. Microenvironmental cues primarily dictate the phenotype and function of mϕ^22^. The repertoire of molecules and factors that trigger mϕ polarization have been well-defined although the field remains highly dynamic^23^. In this work, we sought to understand whether clostridial collagenase, the enzyme used as the wound debridement agent in CSO, may influence the fate of wound inflammation by inducing mϕ polarization.

## Results

### CSO caused a pro-resolution milieu in acute and chronic diabetic wounds

**Figure 1:**
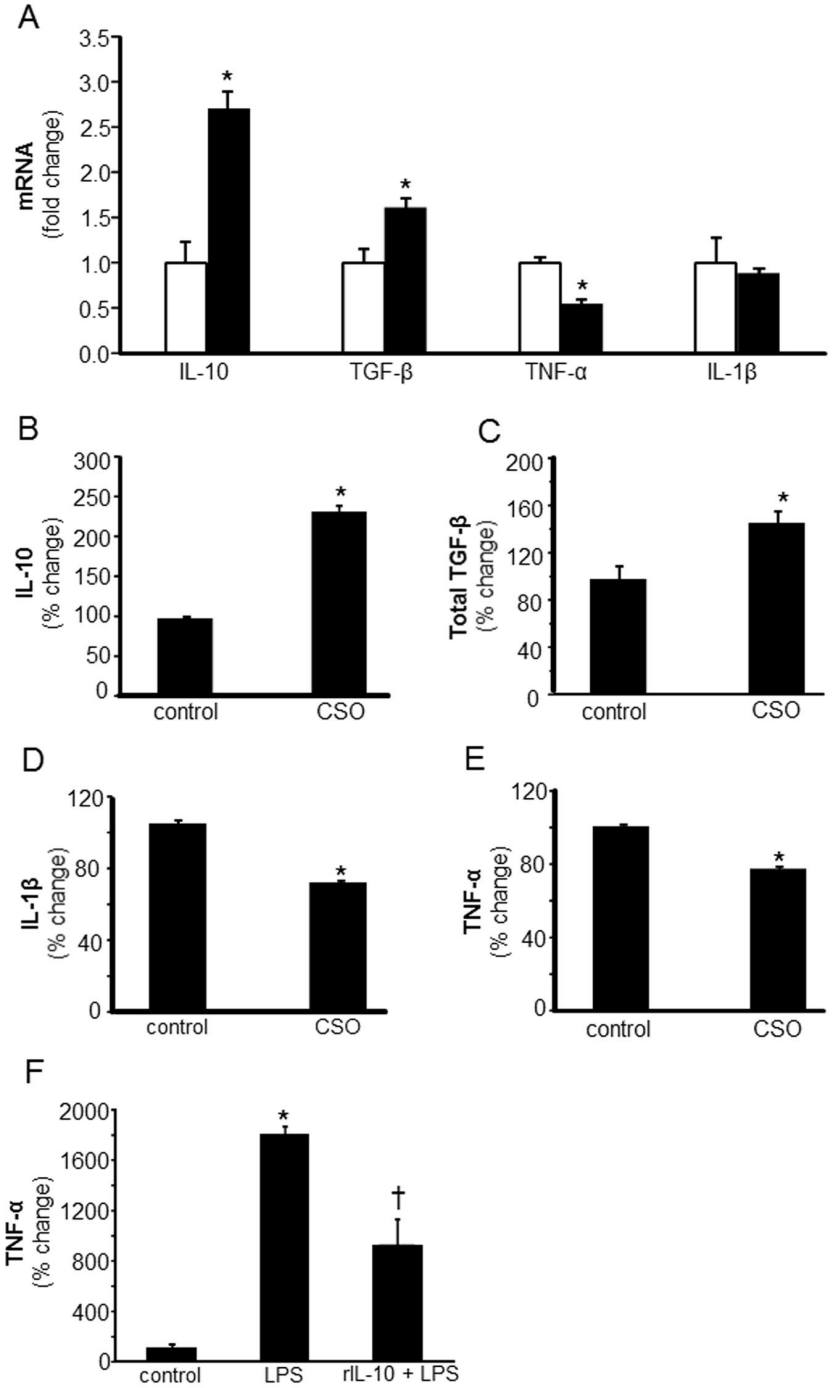
CSO modifies cytokines release from murine wound macrophages contributing towards a pro-resolution milieu in acute wounds. Day 7 wound mϕ were harvested from PVA sponges coated with CSO (300 mg), implanted subcutaneously in C57bl/6 mice. (**A**) Total RNA was isolated and mRNA expression of IL-10, TGF-β, TNF-α and IL-1β was measured using RTPCR. Data are expressed as mean ± SEM (*n* = 5); *p < 0.05 compared to day 7 wound mϕ treated with equivalent amounts of white petrolatum (control). CSO, solid bars; control, blank bars. (**B**–**E**) The isolated wound mϕ treated *in vivo* with CSO (300 mg) were cultured in presence of LPS (1 µg/ml). After 24 h of culture the media was collected and expression of cytokines were measured using ELISA: (**B**) IL-10, (**C**) total TGF-β, (**D**) IL-1β and (**E**) TNF-α. Data are expressed as % compared to white petrolatum (control) treated group, mean ± SEM (*n* = 5); *p < 0.05 compared to day 7 wound mϕ treated with equal amounts of white petrolatum (control) treated with LPS. (**F**) Bone marrow derived macrophages (BMDM) from C57bl/6 mice were treated with recombinant mouse IL-10 for 1 h followed by activation with LPS (1 mg/ml, for 24 h). The release of TNF-α protein in culture media by BMDM was determined using ELISA. Data are mean ± SEM (n = 4); *p < 0.05 compared to control; ^†^*p* < 0.05, compared to LPS treated group.

**Figure 2:**
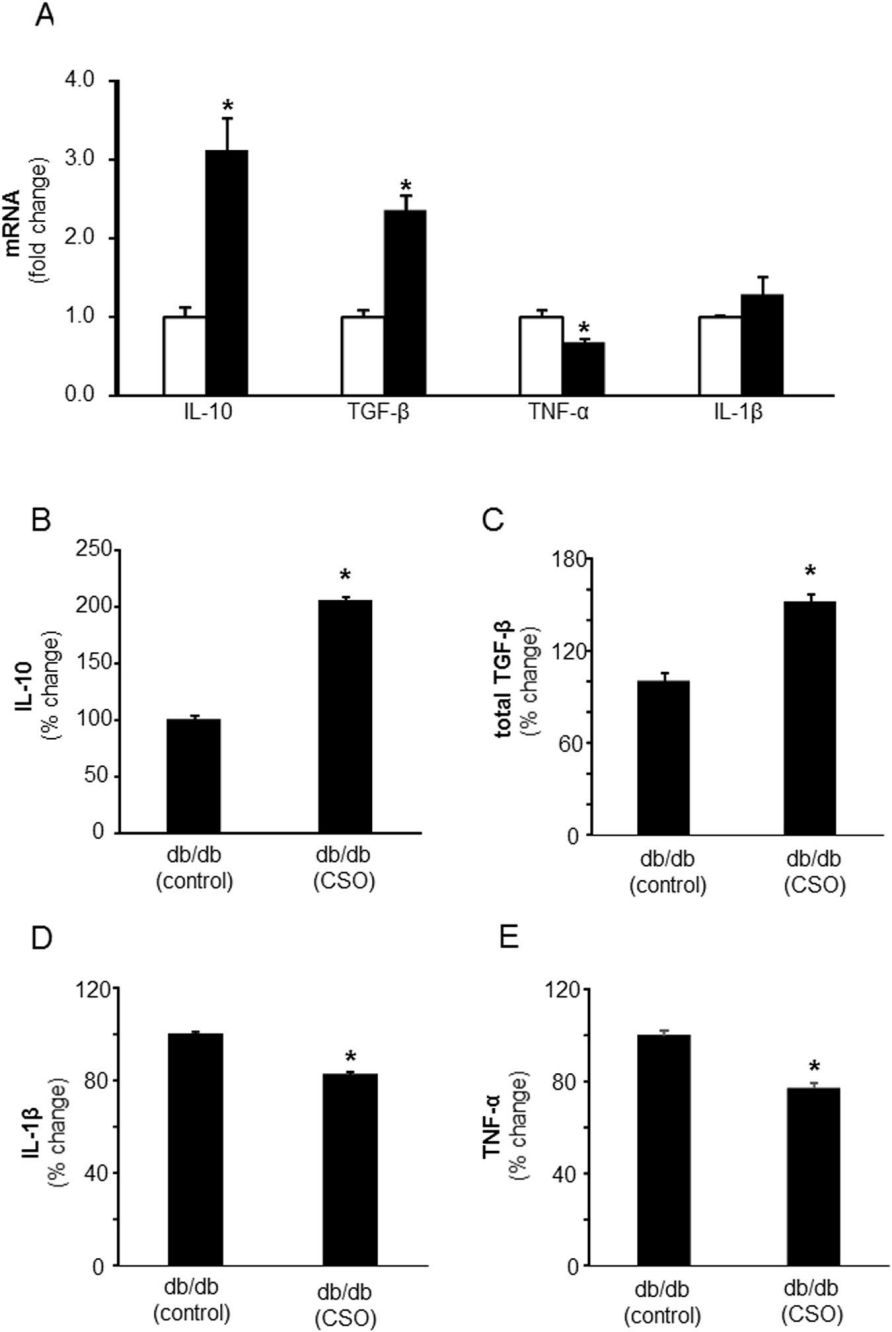
CSO modifies cytokines release from murine wound macrophages contributing towards a pro-resolution *milieu* in chronic diabetic wounds. Day 7 wound mϕ were harvested from PVA sponges coated with CSO (300 mg) implanted subcutaneously in db/db mice. (**A**) Total RNA was isolated and mRNA expression of IL-10, TGF-β, TNF-α and IL-1β was measured using RTPCR. Data are expressed as mean ± SEM (*n* = 5); *p < 0.05 compared to day 7 wound mϕ treated with equivalent amounts of white petrolatum (control). CSO, solid bars; control, blank bars. (**B**–**E**) The isolated wound mϕ treated *in vivo* with CSO (300 mg) were cultured in presence of LPS (1 µg/ml). After 24 h of culture the media was collected and expressions of cytokines were measured using ELISA: (**B**) IL-10, (**C**) total TGF-β, (**D**) IL-1β and (**E**) TNF-α. Data are expressed as % compared to white petrolatum (control) treated group, mean ± SEM (*n* = 5); *p < 0.05 compared to day 7 wound mϕ treated with equal amounts of white petrolatum (control) treated with LPS.

Mounting of a robust inflammatory response followed by a timely resolution are critical components of the healing process^10,16^. Wound mϕ are key players in shaping the wound microenvironment in the inflammatory phase^14,16,24^. To study the effect of CSO on wound microenvironment, mϕ were isolated from wounds treated with CSO or petrolatum (vehicle). Major pro- and anti-inflammatory cytokine/growth factors released by wound mϕ were assessed following potent pro-inflammatory stimulus (lipopolysaccharide, LPS) treatment. Potent upregulation of anti-inflammatory cytokine IL-10 mRNA and protein was noted in CSO-treated group as compared to the control group (Fig. 1A,B). A significant increase in TGF-β mRNA and protein was also observed in CSO-treated group (Fig. 1A,C). The increase in IL-10 and TGF-β was associated with a significant reduction in the levels of TNF-α mRNA and TNF-α/IL-1β proteins (Fig. 1A,D,E). Treatment of bone marrow derived macrophages (BMDM) with recombinant IL-10 significantly inhibited LPS-induced TNF-α release (Fig. 1F) suggesting that increased IL-10 in response to CSO may be implicated in lowering of LPS-induced TNF-α release. To determine whether CSO treatment influenced overall cell flux at the wound-site, we performed multiplexed flow cytometry analysis. While no significant changes in F4/80+ (wmϕ), Ly6G (PMN), or Treg (CD4+ CD25+) cell populations were noted in the CSO treated group, a significant reduction in pro-inflammatory monocytes (CCR2^+^Ly6C^hi^ cells) was observed following CSO treatment (Fig. S1). This finding is supportive of the contention that CSO facilitates an anti-inflammatory pro-resolution *milieu* at the wound-site. Resolution of wound inflammation is known to be impaired in diabetic wounds^15,25^. Interestingly, CSO, in diabetic wounds, promoted the secretion of pro-resolving IL-10 and TGF-β while significantly downregulating TNF-α and IL-1β protein (Fig. 2A–E) demonstrating that CSO may foster pro-resolving *milieu* in the wound microenvironment even under diabetic conditions.

#### CSO induced pro-resolution/healing macrophage (mϕ^heal^) phenotype and attenuated pro-inflammatory (mϕ^inf^) phenotype in acute and chronic diabetic wounds

**Figure 3:**
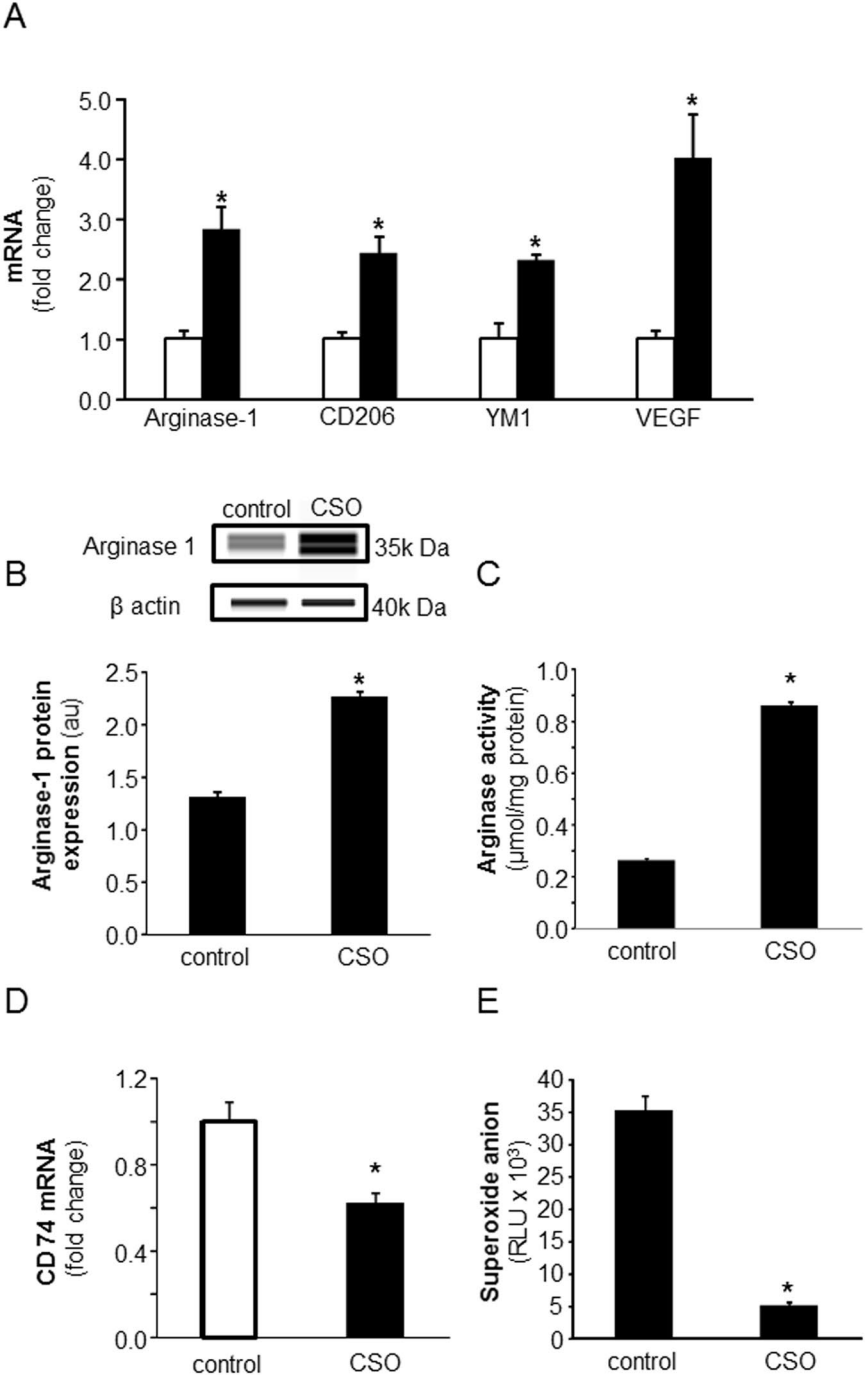
CSO is a potent inducer of mϕ^heal^ macrophage polarization while attenuating M1 phenotype in acute wounds. (**A**) Day 7 wound mϕ were harvested from PVA sponges coated with CSO (300 mg) implanted subcutaneously in C57bl/6 mice. Total RNA was isolated and mRNA expression of Arginase-1, CD206, YM1 and VEGF was measured using RTPCR. CSO, solid bars; control, blank bars. (**B**) Total protein isolated from day 7 wound mϕ of C57bl/6 mice, treated with CSO *in vivo*, was subjected to capillary electrophoresis immunoassay to measure expression of Arginase-1 protein. (**C**) Arginase activity of *in vivo* CSO treated (300 mg) day 7 wound mϕ of C57bl/6 mice were measured by Arginase activity assay kit (Colorimetric). (**D**) mRNA expression of CD74 was measured in day 7 wound mϕ of C57bl/6 mice treated with CSO (300 mg). (**E**) PMA-induced superoxide anion production in day 3 wound mϕ of C57bl/6 mice treated *in vivo* with CSO (300 mg) was measured. For all figure parts, data are expressed as mean ± SEM (*n* = 3–6); *p < 0.05 compared to wound mϕ treated with equal amounts of white petrolatum (control).

**Figure 4:**
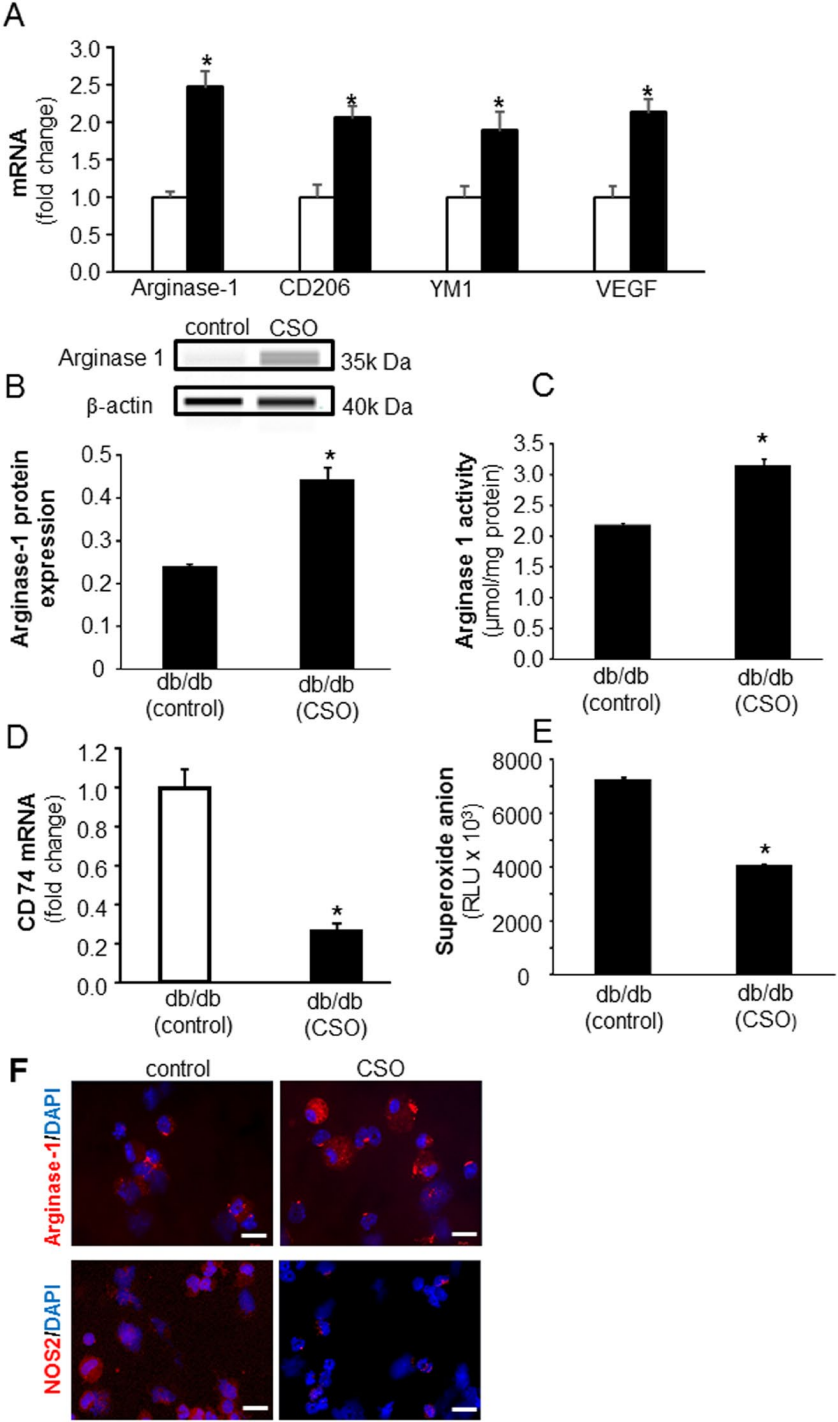
CSO is a potent inducer of mϕ^heal^ macrophage polarization while attenuating M1 phenotype in chronic diabetic wounds. (**A**) Wound mϕ were harvested from PVA sponges coated with CSO (300 mg) implanted subcutaneously in db/db mice on day 7 post-implantations. Total RNA was isolated and mRNA expression of Arginase-1, CD206, YM1 and VEGF was measured using RTPCR. CSO, solid bars; control, blank bars. (**B**) Total protein isolated from day 7 wound mϕ of db/db mice, treated with CSO *in vivo*, was subjected to capillary electrophoresis immunoassay to measure expression of Arginase 1 protein. (**C**) Arginase activity of *in vivo* CSO treated (300 mg) day 7 wound mϕ of db/db mice were measured by Arginase activity assay kit (Colorimetric). (**D**) mRNA expression of CD74 was measured in day 7 wound mϕ of db/db mice treated with CSO (300 mg). (**E**) PMA-induced superoxide anion production in day 3 wound mϕ of db/db mice treated *in vivo* with CSO (300 mg) was measured. For all figure parts, data are expressed as mean ± SEM (*n* = 3–6); *p < 0.05 compared to wound mϕ treated with equal amounts of white petrolatum (control). (**F**) Immunocytochemistry (ICC) images of Arginase-1 (red) and NOS2 (red) protein expression in d7 wound mϕ from db/db animals. Day 7 wound mϕ were harvested from PVA sponges coated with CSO (300 mg) implanted subcutaneously. Counter staining was performed using DAPI (nuclear, blue). Scale bar, 20 μm.

Although debatable, mϕ are commonly classified as pro-inflammatory (mϕ^inf^) *aka* M1 or M(LPS + IFNγ), and pro-reparative (mϕ^heal^) *aka* M2 or M(IL-4) polarization states^16,19,26^. To determine if CSO-induced anti-inflammatory *milieu* at the wound-site is a result of the presence of more pro-reparative mϕ^heal^ in CSO-treated wounds, the expression of mϕ^inf^ and mϕ^heal^ polarization state markers was analyzed using real-time PCR. Analysis of recognized M(IL-4) polarization markers (Arginase-1, CD206, Ym-1, VEGF) revealed a significant increase in M(IL-4) markers in day 7 wound mϕ in the CSO treated group as compared to vehicle-treated (Fig. 3A). Protein expression of Arginase-1, a classical marker for M(IL-4) mϕ^27^, was measured using capillary electrophoresis immunoassay and found to be significantly induced in CSO treatment group (Fig. 3B). Consistent with Arginase-1 mRNA and protein expression, significant increase in the activity of this protein in the CSO-treated group was noted (Fig. 3C). The recognized M(LPS + IFNγ) marker CD74 and PMA-induced superoxide production, on the other hand, were significantly attenuated in wound mϕ exposed to CSO (Fig. 3D,E). Notably, a similar effect of augmentation on mϕ^heal^ markers and attenuation in mϕ^inf^ polarization was also observed in diabetic wounds that are known to have high mϕ^inf^ wound mϕ populations^21,25^ (Fig. 4A–E**)**. Immunocytochemistry (ICC) was performed to determine CSO-induced changes in expression of Arginase-1 and NOS2 protein expression. The data demonstrate that CSO treatment induced Arginase-1 while attenuating NOS2 in majority of the wound mϕ (Fig. 4F).

### The active constituent of CSO facilitated mϕ^heal^ polarization

**Figure 5:**
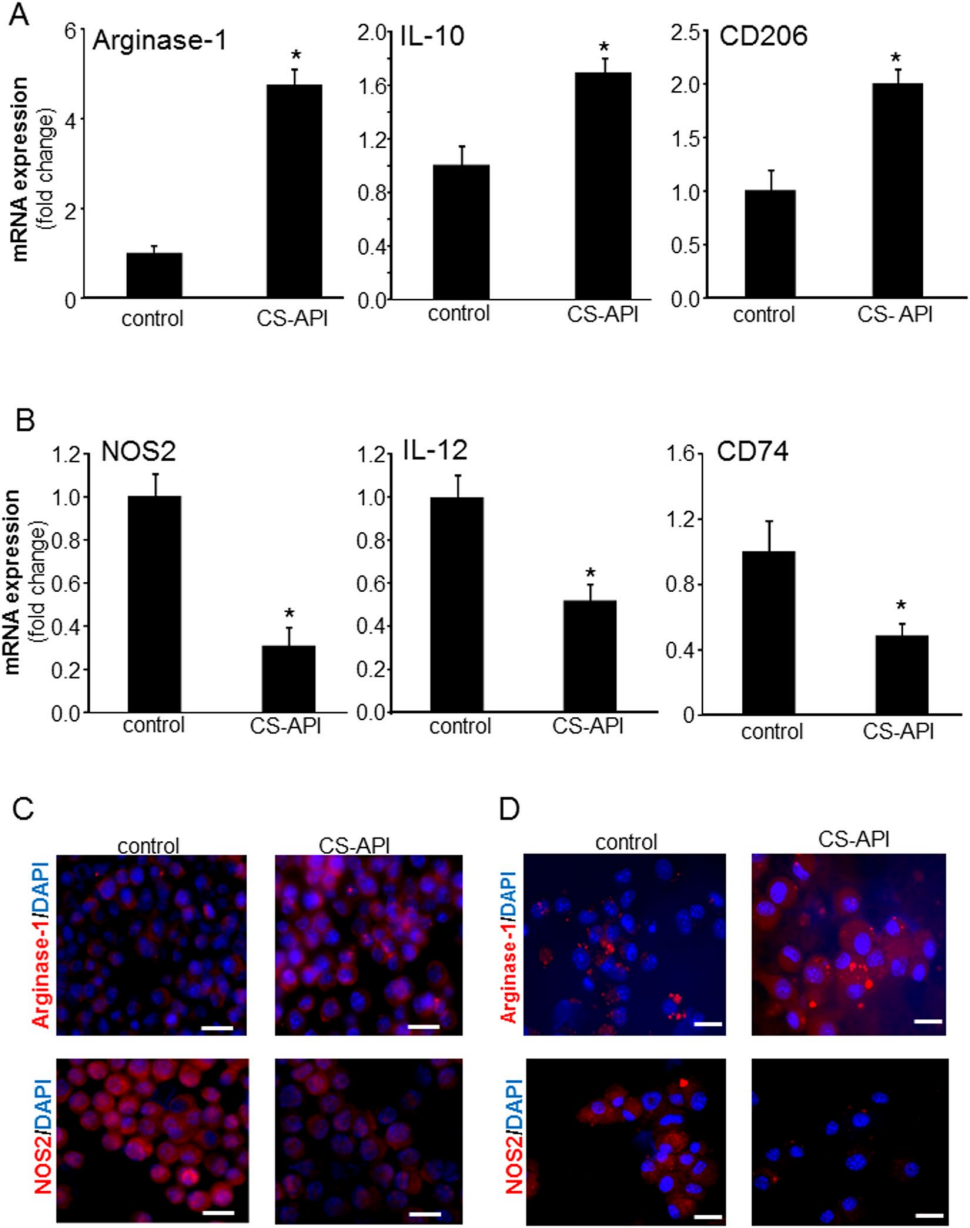
*Ex vivo* treatment with active pharmaceutical ingredient (CS-API) directly induces mϕ^heal^ macrophage polarization while inhibiting the mϕ^inf^ phenotype. (**A**,**B**) Day 3 wound mϕ of C57bl/6 mice were harvested from PVA sponges and treated *ex vivo* with CS-API (250 ng/mL) for 24 hours (A) mRNA expression of Arginase-1, IL-10 and CD206 was measured using RTPCR. Data are expressed as mean ± SEM (*n* = 4); *p < 0.05 compared to day 3 wound mϕ treated with vehicle (control). (**B**) mRNA expression of NOS2, IL-12 and CD74 was measured using RTPCR. Data are expressed as mean ± SEM (*n* = 4); *p < 0.05 compared to day 3 wound mϕ treated with vehicle (control). (**C**,**D**) Immunocytochemistry (ICC) images of Arginase-1 (red) and NOS2 (red) protein expression in (**C**) day 3 wound mϕ of C57bl/6 and (**D**) BMDM from db/db animals treated *ex vivo* with CS-API (250 ng/ml) for 24 h. Counter staining was performed using DAPI (nuclear, blue). Scale bar, 20 μm.

The active ingredients in CSO are clostridial collagenases that are produced by *Clostridium histolyticum* bacteria *via* a proprietary fermentation process^3,8,28,29^. This collagenase material contains two distinct collagenases (col), col G (114 kDa) and col H (110 kDa), and a lesser amount of a non-specific neutral protease (a metalloproteinase, 35 kDa) (data not shown). The active constituent of CSO, henceforth called as CS-API, was used to directly treat isolated wound mϕ *ex vivo* to examine if there is a direct effect of CS-API on mϕ polarization. CS-API exists in a powder form as opposed to CSO that is in ointment form suited for wound applications and not suitable for cell culture application. Based on the cell viability data (>90% viable, data not shown), 250 ng/ml dose was selected for *ex vivo* wound mϕ studies. CS-API directly acted on the wound mϕ and caused polarization to mϕ^heal^ phenotype. Substantial induction in M(IL-4) marker genes, Arginase-1, IL-10 and CD206 in wound mϕ was noted as compared to control group (Fig. 5A). Such induction was concomitant with potent down-regulation of M(LPS + IFNγ), genes, *e.g*. NOS2, IL-12 and CD74 (Fig. 5B). The balance of Arginase-1 and NOS2 protein expression in day 3 wound mϕ and BMDM were determined using ICC (Fig. 5C,D). Increased expression in Arginase-1 and attenuation in the expression of NOS2 by CS-API treatment was noted in both activated wound mϕ as well as naïve BMDM **(**Fig. 5C,D**)**. Next, we determined whether CS-API was effective in influencing polarization in human macrophages. Treatment of human blood monocyte derived macrophages (hMDM) with CS-API resulted in macrophage polarization toward a pro-reparative phenotype as observed with mice wound mϕ and BMDMs. Thus, the anti-inflammatory effect of CSO on macrophage polarization may be generalized across these species (Fig. S2A). Interestingly, CS-API treatment of hMDM, previously polarized towards pro-reparative phenotype, did not further induce the pro-reparative markers IL-10 or Arginase (Fig. S2B). These data recognize that the active component of CSO *i.e*., clostridial collagenases, directly influence mϕ response and function across rodents and humans at the wound-site.

### Molecular mechanisms of CSO mediated promotion of wound m*ϕ*^heal^ polarization

**Figure 6:**
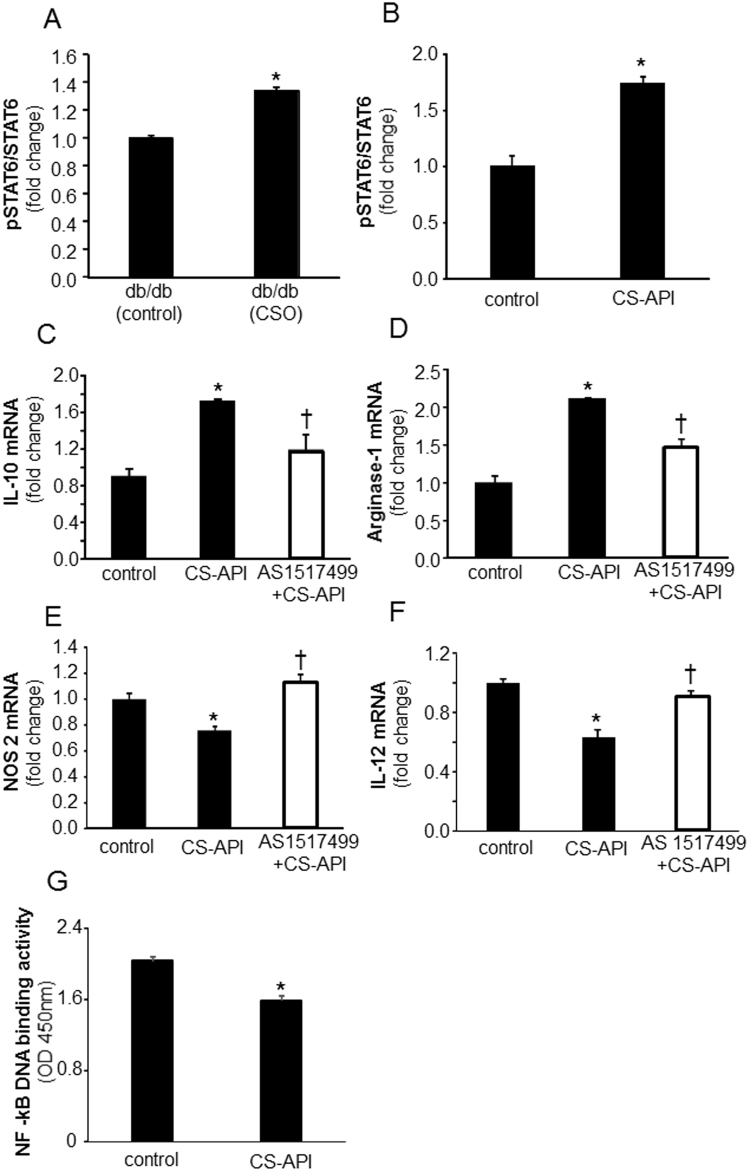
CS-API activates STAT6 while inhibiting NF-κB pathways in macrophage polarization. (**A**) Wound mϕ were harvested from PVA sponges coated with CSO (300 mg) implanted subcutaneously in db/db mice on day 7 post-implantations. Phosphorylation of STAT6 was measured using a cell-based ELISA kit. Data are expressed as mean ± SEM (*n* = 5); *p < 0.05 compared to white petrolatum (control) treated group. (**B**) Cultured mouse mϕ were treated with CS-API (250 ng/ml) for 24 hours. Phosphorylation of STAT6 was measured using a cell-based ELISA kit. Data are expressed as mean ± SEM (*n* = 5); *p < 0.05 compared to control. (**C**–**F**) Cultured mouse mϕ were treated with STAT6 inhibitor AS 1517499 (5 nM) for 1 hour followed by CS-API (250 ng/mL) treatment for 24 hours. mRNA expression of (**C**) IL-10 (**D**) Arginase-1 (**E**) NOS2 and (**F**) IL-12 was measured. Data are expressed as mean ± SEM (*n* = 3–4); *p < 0.05 compared to control, ^†^*p* < 0.05, compared to CS-API treated group. (**G**) Cultured mouse mϕ were treated with CS-API (250 ng/ml) for 24 hours. DNA binding activity of LPS (1 µg/ml, 3 h) inducible NF-κB was measured using an ELISA-based (Trans-AM) method. Data are expressed as mean ± SEM (*n* = 5); *p < 0.05 compared to control.

**Figure 7:**
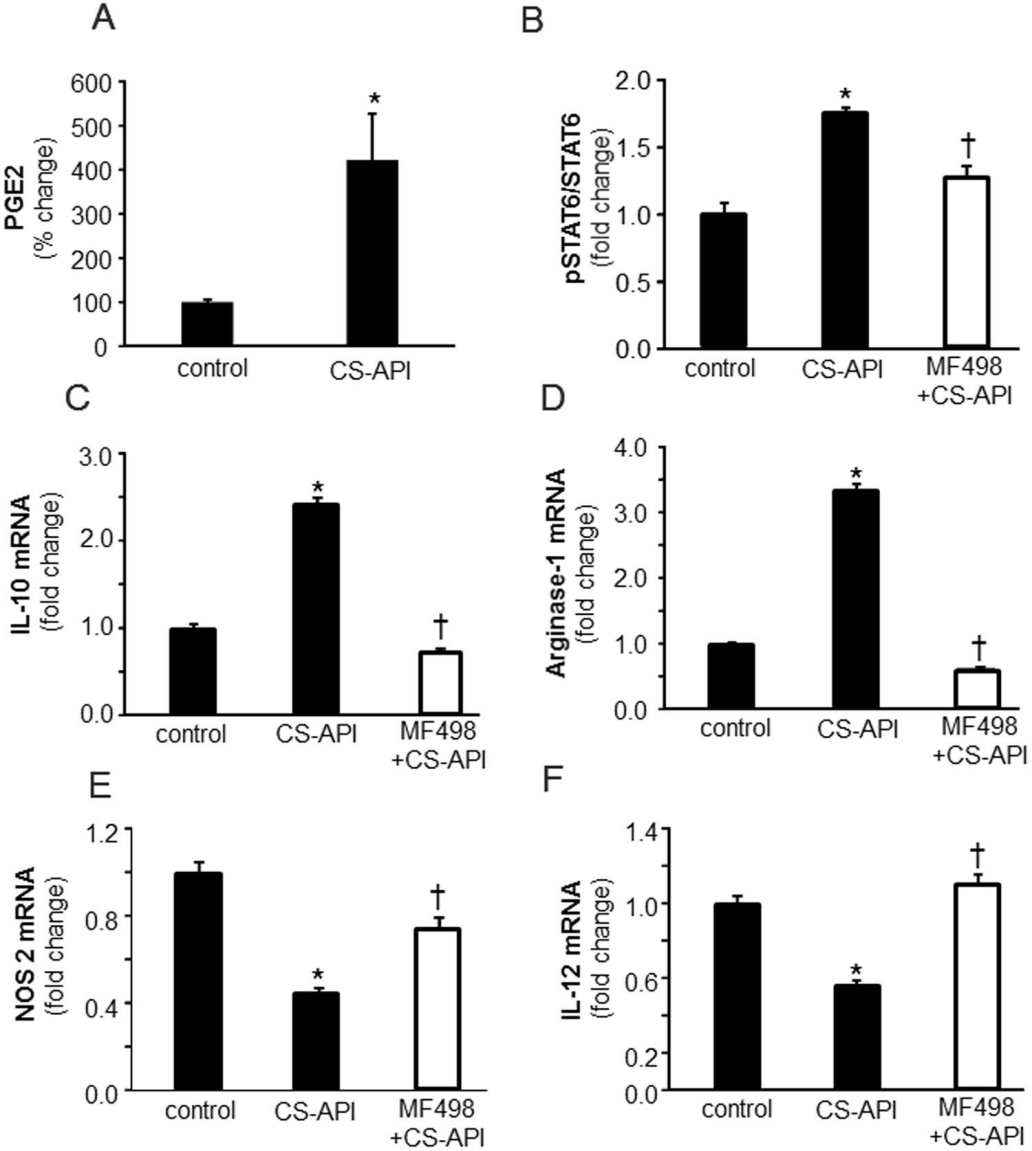
PGE2-EP4 pathway in CS-API mediated STAT6 phosphorylation and macrophage polarization. (**A**) Cultured mouse mϕ were treated with CS-API (250 ng/ml) for 24 hours. PGE_2_ level in the media was measured using ELISA. Data are expressed as mean ± SEM (*n* = 3); *p < 0.05 compared to control. (**B**) Cultured mouse mϕ were treated with EP4 inhibitor MF498 (50 nM) for 1 hour followed by CS-API (250 ng/ml) for 24 hours. Phosphorylation of STAT6 was measured using cell-based ELISA. Data are expressed as mean ± SEM (*n* = 3); *p < 0.05 compared to control, ^†^*p* < 0.05, compared to CS-API treated group. (**C**–**F**) Cultured mouse mϕ were treated with EP4 inhibitor MF498 (50 nM) for 1 hour followed by CS-API (250 ng/ml) for 24 hours. mRNA expression of (**C**) IL-10 (**D**) Arginase-1 (**E**) NOS2 and (**F**) IL-12 was measured. Data are expressed as mean ± SEM (*n* = 3); *p < 0.05 compared to control, ^†^*p* < 0.05, compared to CS-API treated group.

Molecular regulation of mϕ polarization is complex and involves a network of signaling molecules, transcription factors, epigenetic mechanisms, as well as posttranscriptional regulators. Canonical IRF/STAT signaling pathways are activated by IFNs and TLR signaling to skew mϕ function towards M(LPS + IFNγ) phenotype *via* STAT1, while IL-4 and IL-13 treatments skew toward the M(IL-4) polarization/activity *via* STAT6^30^. CSO as well as its active constituent CS-API potently increased phosphorylation of STAT6 both *in vivo* in diabetic wound mϕ and *in vitro* in an established mouse mϕ cell line RAW 264.7 (Fig. 6A,B). To determine if the robust increase in STAT6 phosphorylation by CS-API is the underlying cause for the induction in mϕ^heal^ mϕ polarization, RAW 264.7 mϕ were treated with a specific pharmacological inhibitor of STAT6, AS1517499. AS1517499, or 4-(benzylamino)-2-pyrimidine-5-carboxamide, is a potent and selective inhibitor of STAT6^31^. Pretreatment of mϕ with STAT6 inhibitor abrogated the effect of CS-API on mϕ polarization, eliminating or reducing the shift towards mϕ^heal^ phenotype for different markers (Fig. 6C–F), pointing towards a key role of STAT6 in CS-API and CSO mediated mϕ^heal^ polarization of mϕ at the wound-site. In addition to a key role of STAT6 pathway, a moderate attenuation in LPS inducible NF-κB activation was noted in CS-API treated cultured macrophages (Fig. 6G). In our efforts to further understand the underlying mechanisms, the involvement of the IL-4Rα pathway was studied by blocking IL-4Rα. Although such blocking of IL-4Rα abrogated IL-4 induced Arginase expression, no reduction was noted in CS-API-induced Arginase in the RAW 264.7 mϕ (Fig. S6) ruling involvement of the IL-4-IL-4Rα pathway in CS-API mediated effect on macrophage polarization as unlikely. The classical pathway of STAT6 activation occurs *via* IL-4R. However, alternatives to this canonical pathway for STAT6 activation have been reported^32–40^. To determine if alternative pathways of STAT6 activation are involved in CSO mediated activation of STAT6 → mϕ^heal^ polarization, we screened specific signaling pathways known to induce mϕ^heal^ phenotype. These studies led us to identify a novel PGE_2_-EP4 alternative pathway for CSO-STAT6 activation → mϕ^heal^ phenotype following CS-API treatment (Fig. 7). Treatment of cultured mϕ with CS-API resulted in marked increase in PGE_2_ production **(**Fig. 7A**)**. Our laboratory has earlier reported that PGE_2_ in mϕ acts *via* an EP4–cAMP mediated pathway^41^. Inhibition of PGE_2_ receptor EP4 using specific pharmacological inhibitor (MF498), resulted in significant reduction in CS-API mediated STAT6 activation **(**Fig. 7B**)** as well as a significant induction of mϕ^heal^ phenotype (Fig. 7C–F).

## Discussion

Mϕ are critical mediators of innate immune response and key facilitators of mounting/resolving the wound inflammatory response^16,42^. In response to altering wound microenvironment, considerable plasticity in these cells allows them to acquire and exhibit diverse functional/activation/polarization states^20,43,44^. The factors or cues in the wound microenvironment that drive such diversity are vaguely understood at present. This study provides maiden evidence demonstrating that a commonly used enzymatic wound debridement agent CSO that contains clostridial collagenases as the active ingredient is a powerful inducer of mϕ^heal^ functional state in acute and chronic diabetic wounds. CSO treatment to wounds also promoted a pro-resolving environment that may be beneficial for chronic wounds known to have persistent inflammation as one of the major underlying factors driving the chronicity of these wounds.

The last decade has witnessed a considerable development in understanding of intrinsic mechanisms that regulate the inflammatory response. Such understanding has led to the design of therapies that promote mechanisms favoring resolution of inflammation. Remarkably, a widely used enzymatic debridement agent, CSO, promoted a pro-resolving wound environment by augmenting production of IL-10 by wound-site mϕ in both acute and diabetic chronic wounds. IL-10 is one of the most prominent pro-resolution cytokines that is released by cells of monocytic lineage^45^. The pro-resolution property of IL-10 is attributed to its potent inhibitory effect on the production of a large spectrum of pro-inflammatory cytokines such as IL- 1β, IL-6, and TNF-α^46,47^. Forced overproduction of IL-10 in wounds promoted regenerative adult wound healing by decreasing the inflammatory response^48^. Presence of multifold high levels of pro-inflammatory cytokines is a hallmark of chronic wounds^10,49^ including diabetic ulcers^50,51^. We and others have reported that mϕ from diabetic wounds produce significantly attenuated levels of IL-10 as compared to mϕ from non-diabetic wounds. CSO significantly improved the production of IL-10 in diabetic wound mϕ underscoring the potential to promote resolution of inflammation in chronic diabetic wounds.

Hallmarks of mϕ cell biology include plasticity and dynamicity such that these cells can exist within an inflammatory environment in diverse activation and function states^16,52^. Such diverse activation forms are part of a continuum, the two terminals of this continuum are defined by the classically activated or inflammatory mϕ, and reparative or alternatively activated mϕ^17,52–54^. The classically activated mϕ produces factors that favor a pro-inflammatory *milieu* (mϕ^inf^). These factors include IL–12, TNF-α, IL-6, IL-1β, and nitric oxide (NO) in response to microbial pathogens or LPS^55^. The alternatively activated mϕ are known to favor a pro-resolution and reparative (mϕ^heal^) *milieu* by producing IL-10, TGF-β, Arginase-1 (Arg1), CD206 (mannose receptor) and Clec7a (dectin-1)^55^. The ratio of nitric oxide synthase iNOS to Arg 1 is commonly used to determine the functional status of mϕ^22,56,57^. The M(LPS + IFNγ) & M(IL-4) paradigm represents two contrasting pathways for utilization of an amino acid. Arginine, *via* increased iNOS, in M(LPS + IFNγ) mϕ is metabolized to: arginine →NO + citrulline. In contrast, for M (IL-4) mϕ, arginine → ornithine + urea^58^. NO, a product of iNOS, is a reactive radical, the toxicity of which is greatly enhanced upon reaction with superoxide to form peroxynitrite (ONOO^−^)^59^. Ornithine, generated as a result of arginase metabolism, serves as a substrate for multiple enzymes including: (i) ornithine decarboxylase (ODC), a rate-limiting enzyme in the synthesis of polyamines, and (ii) ornithine aminotransferase (OAT), an enzyme that catalyzes the conversion to proline^27^. Although the role of Arginase in fibrosis has been questioned in regulation of fibrosis^60^, polyamines are essential for cell growth and development and have been shown to exert inhibitory effect against pro-inflammatory cytokines^27^. Proline is essential for the synthesis and maturation of collagen. A critical role of Arginase-expressing and ornithine-producing mϕ^heal^ has been reported in facilitating wound healing^58^. A potent increase in Arginase-expressing mϕ^heal^ in wounds treated with CSO indicates an alternative action of this proven debridement agent in facilitating wound healing. It is now recognized that multiple subtypes of the anti-inflammatory mϕ^heal^ exist at the repair-site^54^. In addition to mϕ^heal^ phenotype that may promote collagen synthesis, collagenolytic subtypes may exist^61,62^. The current study did not subcategorize the mϕ^heal^ at the wound-site.

STAT6 (signal transducers and activators of transcription 6) a member of STAT family of transcription factors^63^, directly regulates transcription of many genes associated with M(IL-4) mϕ phenotype^17,64^. This study noted that CS-API potently activated STAT6 and inhibited NF-κB as a central mechanism to promote M(IL-4) or mϕ^heal^ while attenuating mϕ^inf^ polarization. Abrogating the activation of STAT6 by pharmacological inhibitors resulted in the loss of function of CS-API in facilitating mϕ^heal^ polarization recognizing STAT6 as one of the primary pathways for CSO action on influencing mϕ polarization. STAT6 activation occurs *via* IL-4R mediated canonical^65,66^ as well as non-canonical pathways^32–40^. Our laboratory has earlier reported that PGE_2_ in mϕ acts *via* an EP4–cAMP pathway^41^. PGE_2_ is known to drive mϕ towards mϕ^heal^ polarization *via* a cAMP/CREB pathway^67–69^. Inhibition of PGE_2_ receptor EP4 using specific pharmacological inhibitor (MF498), resulted in significant reduction in CS-API mediated STAT6 activation and a significant induction of mϕ^heal^ phenotype. These new data implicate a novel alternative PGE_2_-EP4 pathway for STAT6 activation and a significant induction of mϕ^heal^ phenotype following CS-API treatment.

The NF-κB family of transcription factors regulate the expression of numerous genes implicated in immunity and inflammation^70^.We have previously reported a novel miR-21-NFκB-PTEN mediated pathway in the regulation of wound macrophage polarization^46^.

The principle active ingredients in CSO are collagenases derived from a specific propriety strain of *Clostridium histolyticum*^3,8^. First reported in 1953, clostridial collagenases are encoded by col G and col H genes for class I and class II type of collagenases, respectively^71^. Unlike mammalian matrix metalloproteinases (MMPs) that cleave native collagen into specific three-quarter and one-quarter fragments, clostridial collagenases breakdown repeating Gly-X-Y collagen sequences at distinct Y-Gly bond containing sites^71^. A recent study has identified novel collagen fragments, and collagen-associated peptides derived from thrombospondin-1, multimerin-1, fibronectin, and tenascin-C, generated from CSO-digested human dermal capillary endothelial and fibroblastic extracellular matrix (ECM)^8^. It is plausible that such degradation products of CSO are implicated in the mechanism of action reported in this study. Additionally, a direct effect of CS-API on mϕ receptors/pathways leading to induction of mϕ^heal^ polarization may not be ruled out. Taken together, this work sheds new light on what is currently utilized as a standard wound debridement agent in clinics. Findings of this work reposition CSO as a potential agent that may be effective in resolving wound inflammation, including wounds of diabetics, *via* STAT6 and NF-κB pathways.

## Materials and Methods

### Isolation of murine wound m*ϕ* and bone marrow-derived macrophages (BMDM)

All animal studies have been approved by, and all methods were performed in accordance with the guidelines and regulations set by The Ohio State University’s Institutional Animal Care and Use Committee (IACUC). Male C57BL/6 mice (8–12 weeks old) were obtained from Harlan Laboratories (Envigo). Mice homozygous for spontaneous mutation of the leptin receptor (Leprdb) (BKS.Cg-m +/+ Leprdb/J, or db/db; stock no 000642) were procured from Jackson Laboratories. For wound mϕ isolation, circular (8 mm) sterile PVA sponges loaded with (300 mg/sponge) CSO (Smith & Nephew, Inc., Fort Worth, TX) or white petrolatum vehicle (control) (Fougera Pharmaceuticals Inc., NY) were implanted subcutaneously on the backs of 8 to 12 week-old mice^15^. The incisional wounds were sutured and closed after implantation of the PVA sponges. Sponge-infiltrated wound mϕ were isolated after 3 or 7 days post-implantation, as previously described^15,25,41^. In brief, following induction of anesthesia, subcutaneously implanted PVA sponges were harvested and wound cell suspension was generated by repeated compression of the sponges. The cell suspension was then subjected to magnetic cell sorting using mouse anti-CD11b tagged microbeads (Miltenyi Biotec, Auburn, CA). This procedure yields a purified (>95%) population of wound mϕ as determined by F4/80 staining^15,25,41^. Murine bone marrow-derived macrophages (BMDM) were isolated as previously described^41^.

### ELISA

Levels of cytokines secreted by wound mϕ were measured using commercially available ELISA kits^41,46^. For the assay, wound mϕ isolated from CSO or vehicle treated groups were seeded in 6-well or 12-well plates followed by activation with LPS (1 µg/ml) for 24 h. After 24 h, the media was collected and the cytokine levels in culture media were measured using commercially available ELISA kits (R & D Systems, Minneapolis, MN) as per manufacturer’s instructions.

### Isolation of RNA, reverse transcription and quantitative RT-PCR (qRT-PCR)

mirVana RNA isolation kit (Ambion, Austin, TX), was used according to the manufacturer’s instructions to extract total RNA as described^72^. Gene expression was measured by real-time qPCR assay using DNA intercalation dye SYBR Green-I (Applied Biosystems, CA) as described previously^15,25,41,46,73,74^.

### Arginase activity assay

Arginase activity was determined with Arginase activity assay kit per manufacturer’s recommendation (Abcam, Cambridge, MA). In this assay, Arginase reacts with arginine through an intermediary product reacts with the OxiRed™ Probe to generate a colored product that can be read at OD 570 nm. For the assay, cell lysates or standards are mixed with a reaction cocktail provided with the kit followed by incubation at 37 °C for 30 mins. The colored product was read at 570 nm with a Plate Reader. The activity was normalized using equal amount of proteins in the cell lysate. The activity was expressed in μmol/mg of protein.

### Superoxide anion production

Superoxide anion production was measured using a standard luminol based LumiMax Superoxide anion detection kit (Agilent technologies, Santa Clara, CA)^72^. The principle of the assay is that superoxide anion oxidizes luminol in a reaction that produces photons of light that are readily measured with a standard luminometer.

### Capillary Electrophoresis Immunoassay

Capillary electrophoresis immunoassay was performed using the Simon (Protein Simple, Santa Clara, CA) according to the manufacturer’s protocol. The sample loading, electrophoresis and immunodetection was performed using a fully automated capillary electrophoresis system as described^75^. Capillary electrophoresis immunoassay was carried out at room temperature, and using default instrument settings (stacking/sample load time, 12 sec/8 sec; separation time, 40 min; blocking time, 15 min; primary antibody, 120 min; secondary antibody, 60 min). Arginase-1 (BD Biosciences, CA; dilution 1:30), and β-actin (Sigma Aldrich, MO; dilution 1:100) primary antibody were used for immuno-detection. The digital images were analyzed using Compass software (Protein Simple).

### Immunocytochemistry

Cytospin smears from wound mϕ suspension were fixed in cell fixation buffer (BD Cytofix, BD Biosciences, CA) for 10 min. Following fixation, wound mϕ were washed, blocked in 10% NGS for 30 min and were incubated in primary antibodies for Arginase-1 (1:100; BD Biosciences) and iNOS (1:100; Abcam,). Fluorescence tagged secondary antibody detection was performed with Alexa Fluor 568 secondary antibody (1:200, Life Technologies) as described previously^25^.

### Identification of wound cell populations by flow cytometry

Cytometry markers used to identify leukocyte subsets included: Alexa Fluor® 488-F4/80, PerCP-Ly6G, APC-Ly6C, PE-CCR2 (Biolegend), PE-CD4 and FITC-CD25 (Miltenyi). Cells were surface-stained for 30 minutes in staining buffer (1 × DPBS/1% BSA) as described previously^43^. Cells were then gated using forward and side scatter characteristics for leukocytes with at least 2,000 gated events recorded using BDTM LSR II flow cytometry (BD Biosciences) and analyzed using FlowJo and BD FACSDIVA™ software.

### Cell based ELISA study

STAT6/pSTAT6 levels in mϕ were measured using commercially available cell-based ELISA kits as per manufacturer’s instructions. (R & D Systems and Abcam).

### DNA binding of NF-κB

Nuclear protein extracts from mϕ were prepared using the nuclear protein extraction kit (Active Motif, Carlsbad, CA). Binding of NF-κB family of proteins to their consensus sites was determined using an ELISA-based Trans-AM NF-κB kit (Active Motif, Carlsbad, CA) following manufacturer’s instructions as previously described^46^.

### Statistics

Data are reported as mean ± SEM as indicated in the respective figure legends. Student’s *t* test (two-tailed) was used to determine significant differences. Comparisons among multiple groups were tested using ANOVA, and *p* < 0.05 was considered statistically significant.

### Disclosures

The authors declare that CSO, CS-API and partial research funding was provided by Smith & Nephew, Inc. ER and LS are current employees of Smith & Nephew, Inc (R&D, Fort, Worth).

## Electronic supplementary material

Supplementary information
