# Supplementary material for: Novel mechanisms of Collagenase Santyl Ointment (CSO) in wound macrophage polarization and resolution of wound inflammation
Source: Sci Rep. 2018 Jan 26;8:1696. doi: 10.1038/s41598-018-19879-w (PMC5786052; doi:10.1038/s41598-018-19879-w)
Supplement: Supplementary file 1 — Supplementary information [file 41598_2018_19879_MOESM1_ESM.pdf]

NOVEL MECHANISMS OF COLLAGENASE SANTYL OINTMENT (CSO) IN WOUND MACROPHAGE POLARIZATION AND RESOLUTION OF WOUND INFLAMMATION

Amitava Das[1†], Soma Datta[1†], Eric Roche[2], Scott Chaffee[1], Elizabeth Jose[1], Lei Shi[2], Komel Grover[2], Savita Khanna[1], Chandan K. Sen[1] and Sashwati Roy[1*]

[1]Department of Surgery, Center for Regenerative Medicine and Cell Based Therapies and Comprehensive Wound Center, The Ohio State University Wexner Medical Center, Columbus, OH, 43210, USA
[2]Research & Development, Smith & Nephew, Inc., Fort Worth, Texas, USA

[†]equally contributed to work

Running Title: Collagenase in resolution of inflammation

Address correspondence to:
Sashwati Roy, PhD
473 West 12th Avenue, 511 DHLRI
The Ohio State University Medical Center
Columbus, Ohio 43210
Tel. 614 247 7658; Fax 614 247 7818
E.mail: sashwati.roy@osumc.edu

SUPPLEMENTARY FIGURES

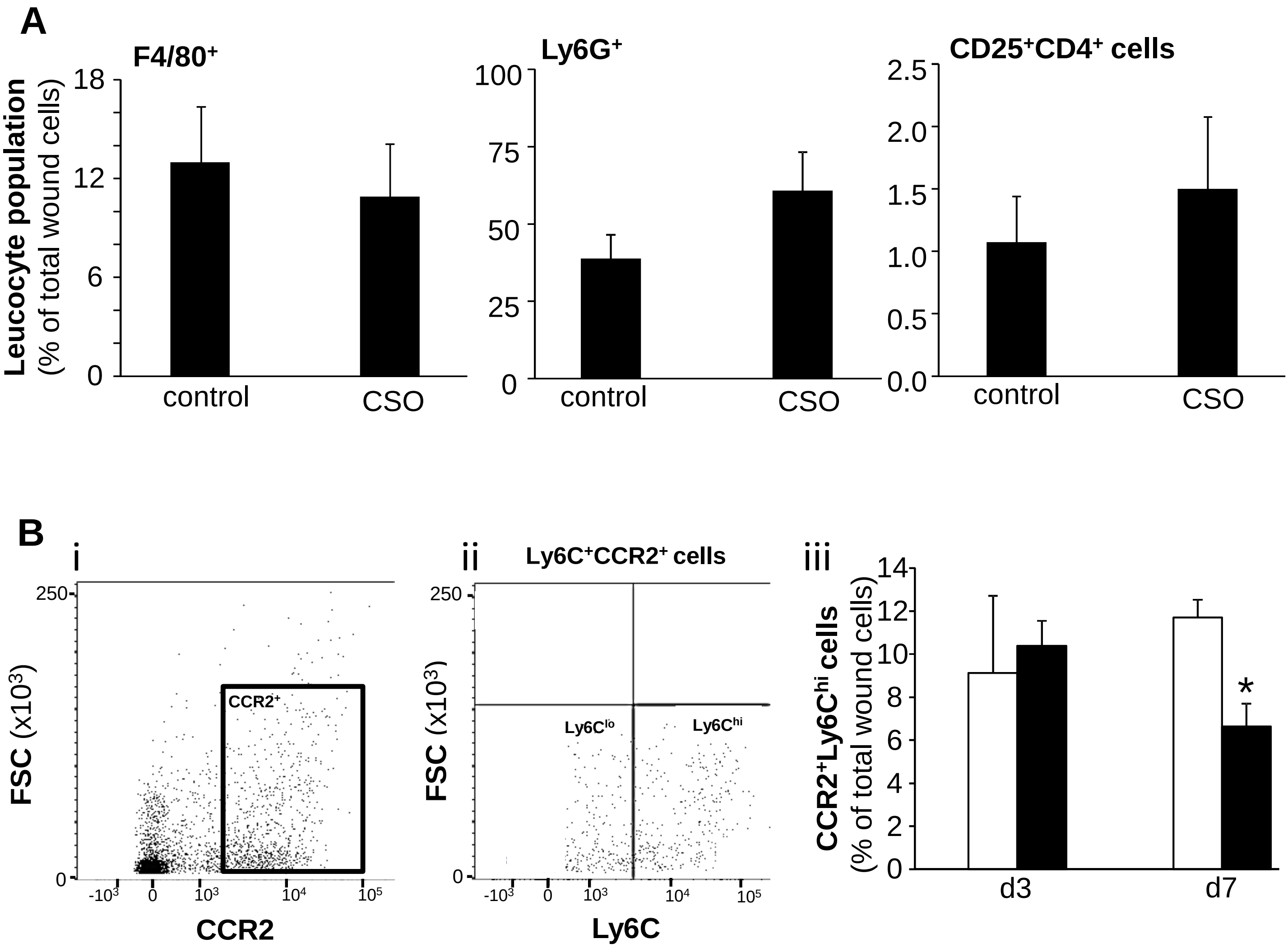
A
F4/80+
Ly6G+
CD25+CD4+ cells
Leucocyte population
(% of total wound cells)
18
12
6
0
100
75
50
25
0
2.5
2.0
1.5
1.0
0.5
0.0
control
CSO
B
i
ii
iii
Ly6C+CCR2+ cells
250
0
FSC (x10³)
CCR2+
Ly6Clo
Ly6Chi
-10³
0
10³
10⁴
10⁵
CCR2
Ly6C
CCR2+Ly6Chi cells
(% of total wound cells)
14
12
10
8
6
4
2
0
*
d3
d7

Figure S1

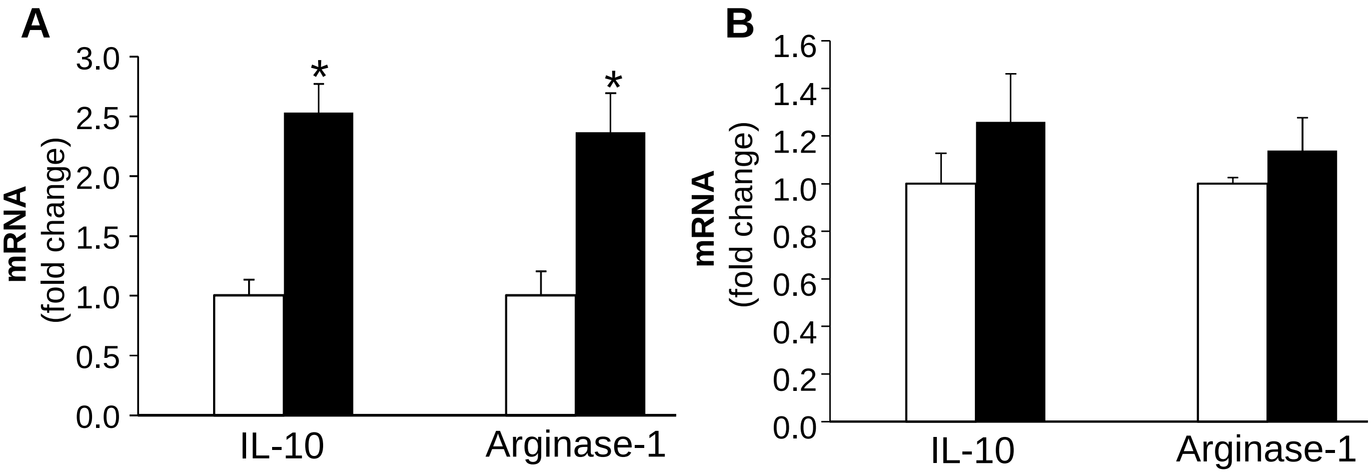
A
B
mRNA
(fold change)
3.0
2.5
2.0
1.5
1.0
0.5
0.0
*
*
IL-10
Arginase-1
1.6
1.4
1.2
1.0
0.8
0.6
0.4
0.2
0.0

Figure S2

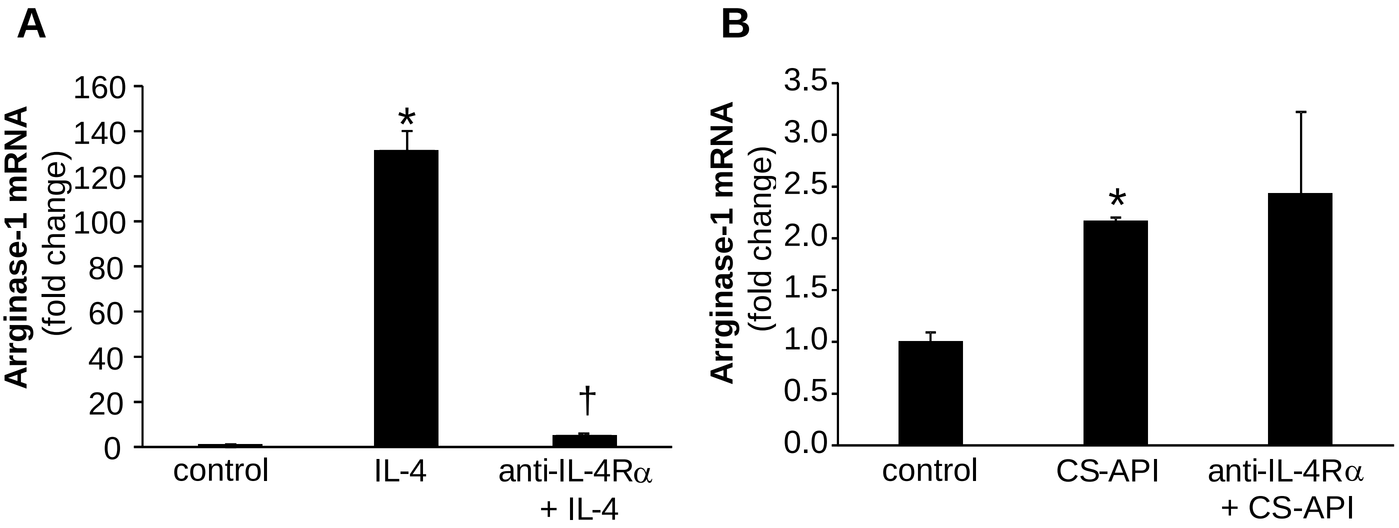
A
Arginase-1 mRNA
(fold change)
160
140
120
100
80
60
40
20
0
*
†
control
IL-4
anti-IL-4Rα
+ IL-4
B
Arginase-1 mRNA
(fold change)
3.5
3.0
2.5
2.0
1.5
1.0
0.5
0.0
*
control
CS-API
anti-IL-4Rα
+ CS-API

Figure S6

Supplementary Figures.

Figure S1. Flow cytometry analysis of wound cell populations following CSO treatment. Wound cells were harvested from PVA sponges coated with CSO (300 mg), implanted subcutaneously in C57bl/6 mice and were immunostained with leukocyte markers and subjected to flow cytometry analysis. (**A**) d7 leukocyte populations F4/80$^+$, Ly6G$^+$ and CD25$^+$CD4$^+$ were quantified from the gated cell populations. (**B**) d3 and d7 populations of CCR2$^+$Ly6C^{hi} cells. **i**) CCR2$^+$ (PE positive) cells were gated against relevant IgG control; **ii**) scatter blot analysis of Ly6C$^+$/CCR2$^+$ gated cells to determine the expression Ly6C^{hi} or Ly6C^{lo} cells. iii) quantification of cells shown in ii. CSO, solid bars; control, blank bars. Data are mean ± SEM (n = 5-8); **p*<0.05 compared to d7 wound mϕ treated with equal amounts of white petrolatum (control).

Figure S2. CSO induces macrophage polarization to a pro-resolving phenotype in human blood monocyte derived macrophages (hMDM) but not in pro-resolving (M IL-4) polarized hMDM. A. hMDM were treated with CS-API (250ng/ml) for 24h. Total RNA was isolated and IL-10 and Arginase-1 mRNA expression was measured using RTPCR. Data are expressed as mean ± SEM (n=4). **p*<0.05 compared to control. **B.** hMDM were polarized to pro-resolving {M(IL-4)} macrophages using IL-4 (20ng/ml) and IL-13 (20ng/ml) for 48h and then treated with CS-API (250ng/ml) for 24h. Total RNA was isolated and mRNA expression of IL-10 and Arginase-1 was measured using RTPCR. CSO, solid bars; control, blank bars. Data are expressed as mean ± SEM (n=4). **p*<0.05 compared to control.

Figure S6. CS-API mediated macrophage polarization is independent of classical IL4- IL4R activation pathway. Cultured mouse mϕ were treated with IL-4R-α neutralizing antibody (10µg/ml) for 1 hour followed by **(A)** IL-4 (20ng/ml) or **(B)** CS-API (250 ng/mL) treatment for 24 hours. Arginase-1 mRNA expression was measured using RTPCR. Data are expressed as mean ± SEM (*n*=3); *$p<0.05$ compared to control, $^{\dagger}p<0.05$, compared to IL-4 treated group.
